# Increased Osteocyte Lacunae Density in the Hypermineralized Bone Matrix of Children with Osteogenesis Imperfecta Type I

**DOI:** 10.3390/ijms22094508

**Published:** 2021-04-26

**Authors:** Matthias Mähr, Stéphane Blouin, Martina Behanova, Barbara M. Misof, Francis H. Glorieux, Jochen Zwerina, Frank Rauch, Markus A. Hartmann, Nadja Fratzl-Zelman

**Affiliations:** 1 1st Medical Department, Ludwig Boltzmann Institute of Osteology at the Hanusch Hospital of OEGK and AUVA Trauma Centre Meidling, Hanusch Hospital, 1140 Vienna, Austria; matthias.maehr@humanitas.co.at (M.M.); stephane.blouin@osteologie.lbg.ac.at (S.B.); Martina.Behanova@osteologie.lbg.ac.at (M.B.); barbara.misof@osteologie.lbg.ac.at (B.M.M.); Jochen.Zwerina@osteologie.lbg.ac.at (J.Z.); Markus.Hartmann@osteologie.lbg.ac.at (M.A.H.); 2 Genetics Unit, Shriners Hospital for Children and McGill University, Montreal, ON H4A 0A9, Canada; glorieux@shriners.mcgill.ca (F.H.G.); frank.rauch@mcgill.ca (F.R.)

**Keywords:** osteogenesis imperfecta, bone, transiliac, histomorphometry, osteocytes, matrix mineralization, children

## Abstract

Osteocytes are terminally differentiated osteoblasts embedded within the bone matrix and key orchestrators of bone metabolism. However, they are generally not characterized by conventional bone histomorphometry because of their location and the limited resolution of light microscopy. OI is characterized by disturbed bone homeostasis, matrix abnormalities and elevated bone matrix mineralization density. To gain further insights into osteocyte characteristics and bone metabolism in OI, we evaluated 2D osteocyte lacunae sections (OLS) based on quantitative backscattered electron imaging in transiliac bone biopsy samples from children with OI type I (n = 19) and age-matched controls (*n* = 24). The OLS characteristics were related to previously obtained, re-visited histomorphometric parameters. Moreover, we present pediatric bone mineralization density distribution reference data in OI type I (*n* = 19) and controls (*n* = 50) obtained with a field emission scanning electron microscope. Compared to controls, OI has highly increased OLS density in cortical and trabecular bone (+50.66%, +61.73%; both *p* < 0.001), whereas OLS area is slightly decreased in trabecular bone (−10.28%; *p* = 0.015). Correlation analyses show a low to moderate, positive association of OLS density with surface-based bone formation parameters and negative association with indices of osteoblast function. In conclusion, hyperosteocytosis of the hypermineralized OI bone matrix associates with abnormal bone cell metabolism and might further impact the mechanical competence of the bone tissue.

## 1. Introduction

In recent years, the central role of osteocytes in orchestrating skeletal metabolism has been gradually revealed. Osteocytes are the most abundant and longest-living bone cells, capable of sensing and responding to mechanical loading, secreting hormones regulating bone mineral metabolism and factors directing bone formation and resorption [1,2,3,4]. They function as regulators of bone matrix mineralization and coordinators of osteoblast and osteoclast activity through the secretion of diverse signal proteins such as phosphate-regulating gene with homologies to endopeptidases on the X chromosome (PHEX), dentin matrix acidic phosphoprotein 1 (DMP1), Sclerostin (SOST), Dickkopf-1 (Dkk-1), receptor activator of nuclear factor β ligand (RANKL) and osteoprotegerin (OPG). Moreover, osteocytes also affect distant organs such as kidney through the secretion of fibroblast growth factor 23 (FGF23) and muscle through WNT1, WNT3A and prostaglandin E2 (PGE_2_) [5,6,7]. Osteocytes derive from matrix-producing polygonal osteoblasts that undergo a differentiation process, called osteocytogenesis, towards stellate cells that reside in the mineralized matrix within lacunar spaces. This transformation is characterized by a marked decrease in cell body volume and formation of multiple cytoplasmic processes running through a network of canaliculi connected to bone surface, bone marrow and blood vessels [2,3]. There is a growing body of evidence that bone homeostasis does not only depend on osteocyte function but also on osteocyte number, viability and connectivity [4]. Conversely, aging, inflammatory processes, metabolic and genetic disorders that affect the skeleton and, in particular, osteogenesis imperfecta may alter osteocyte size, number and function [8,9,10]. 

Osteogenesis imperfecta (OI) is phenotypically and genetically a heterogeneous heritable bone dysplasia, affecting 1 in 20,000 births, with its hallmark features of low bone mass and increased bone fragility [11,12,13]. “Classical” OI is caused by mutations in genes encoding type I procollagen α-chains that result in different clinical severities from mild to lethal. OI type I is the mildest and the most frequently occurring type of the dysplasia and results from two different forms of mutations: qualitative and quantitative mutations [14]. Qualitative point mutations generally affect a glycine residue in one of the two genes encoding collagen α-chain, *COL1A1* or *COL1A2*. As a result, a mixture of normal and abnormal collagen is being incorporated into the bone matrix [13]. Quantitative mutations arise from nonsense or frameshift mutations, generally in one *COL1A1* allele [15]. As the α1 chains produced from the intact allele have a normal amino acid sequence, the resulting collagen structure is not altered but the secreted amount is reduced by 50% [16]. 

Despite the importance of osteocytes for bone matrix mineralization and homeostasis, surprisingly little is known about the specific implication of osteocytes in the pathogenesis of OI [17]. It has been observed that lacunae density is increased in OI bone, but this has been quantified only in very few patients [18,19,20,21]. Although increased lacunae porosity in bone from children with OI was not found to impact mechanical properties [21,22,23], it reflects an altered lacuno-canalicular network architecture that may negatively influence the mechano-sensing [24] and, thus, potentially contribute to bone fragility [19,25]. In addition, osteocytes sense mechanical stimuli through interaction with the surrounding bone matrix, which is also profoundly altered in OI [20,26]. Beyond the collagen abnormalities, non-collagen components of the organic matrix, such as the relative number of proteoglycans, are reduced, while the mineral content of the matrix is increased, which makes the matrix stiffer and more brittle [12,27,28,29]. 

Moreover, several osteocyte-directed pathways that contribute to bone turnover are disturbed in OI. Osteocytes in OI produce high levels of RANKL, a cytokine that mediates osteoclastogenesis [30] and, hence, higher bone resorption is observed both in mouse models and in patients [30,31,32]. Furthermore, the Wnt/β-catenin signaling pathway, a major regulator of osteoblastogenesis and bone formation is suspected to be dysregulated in OI [10]. Increased expression of DKK1 and sclerostin, two glycoproteins secreted by the mature osteocytes and major inhibitors of the Wnt/β-catenin pathway are thought to contribute to the low bone mass in OI. It was recently shown that serum levels of DKK1 are indeed elevated in children with OI [32]. While sclerostin serum levels were found normal or even decreased in OI patients [33,34,35], treatment with sclerostin antibody increased bone mass and improved bone mechanical strength in diverse mouse models and in patients [36,37,38]. In addition, the profoundly perturbed osteoblasts in OI might also sense and react differently to sclerostin or other local factors. Indeed, misfolded and truncated pro-collagen molecules accumulate within the endoplasmic reticulum (ER) and trigger stress responses in osteoblasts with activation of intracellular degradation pathways and increased cell death [39]. Moreover, delayed triple-helix formation due to the OI mutation leads to over-modification of the collagen by the ER-resident lysyl- and prolyl-hydroxylases and to reduced collagen secretion [12,40,41,42]. Finally, not only the osteocyte number but also the osteoblast number is abnormally high in OI bone, although the amount of matrix deposited is markedly low [19]. Thus, this would suggest that the increased osteocyte lacunae density might be related to the increased osteoblast number. 

In the present study, we reanalyzed bone biopsy samples from healthy children and children with OI type I, previously investigated for standard histomorphometry [19,43] and bone matrix mineralization by quantitative backscattered electron imaging [44,45]. Here, we measured 2D-osteocyte lacunae density, size and shape, using a field emission scanning electron microscope. This method had already been applied for the characterization of osteocyte lacunae in OI type V, a distinct form of OI, characterized by exuberant primary bone formation and hypercellularity resembling woven bone [46,47,48,49]. We, thus, also compared osteocyte lacunae parameters between OI type I and OI type V bone. Our second aim was to assess the relationship between parameters of osteocyte lacunae and re-visited bone histomorphometry parameters obtained in the identical biopsy samples from OI type I patients and healthy controls. Third, we established new reference data for bone mineralization density distribution using the field emission scanning electron microscope to complement the existing reference data measured on a different device reported previously [44,45]. 

## 2. Results

### 2.1. Osteocyte Lacunae Density but Not Size Is Increased in OI Type I Bone Compared to Healthy Controls

**Figure 1:**
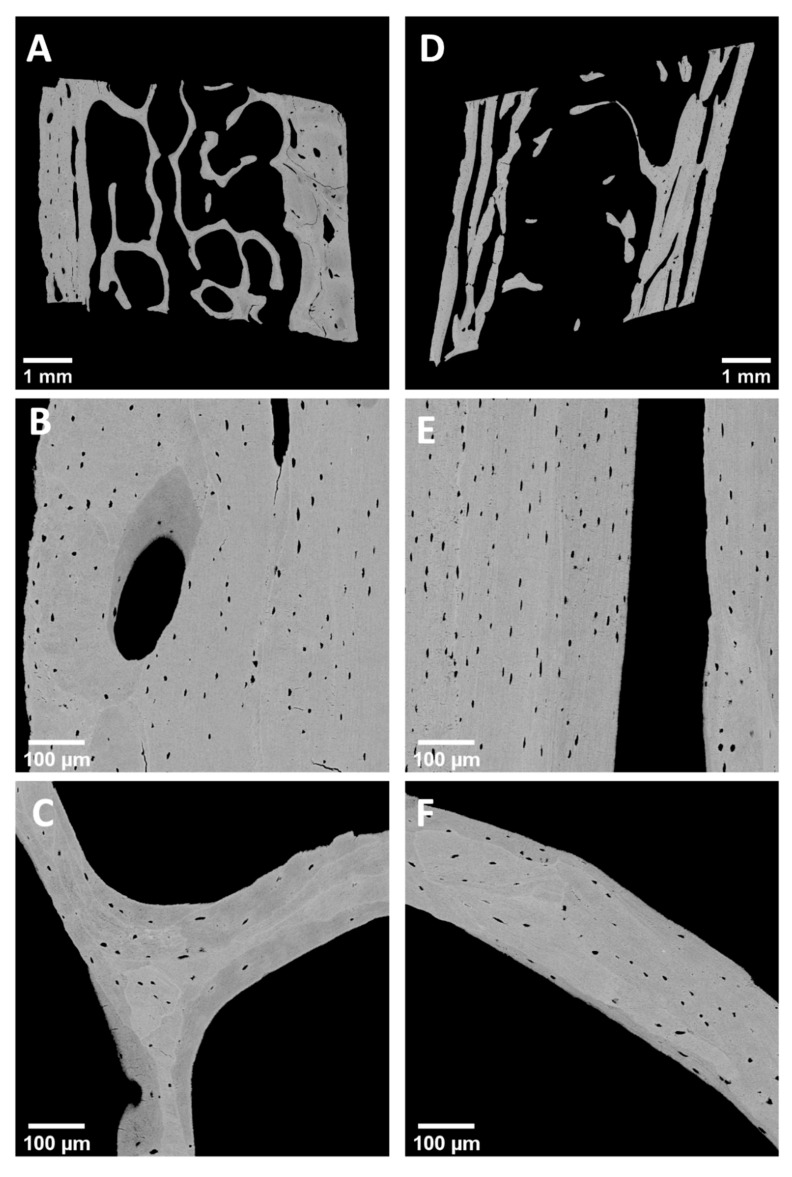
Example of quantitative backscattered images of transiliac bone biopsy samples from healthy children (**A**–**C**) and children with OI type I (**D**–**F**). Typical backscattered electron images of a complete biopsy samples (**A**) from a 2-year-old healthy boy and (**D**) from a 7.6-year-old female OI type I patient with qualitative mutation. Note the decreased number and thickness of trabeculae and the decreased cortical width in OI type I bone. Example of quantitative backscattered images used for OLS analysis of cortical bone (**B**,**E**) and trabecular bone (**C**,**F**). Note the increased osteocyte lacunar density that can be judged especially in the cortical compartment of OI type I bone (**B** versus **E**). Nominal magnification: (**A**,**D**): 65×; (**B**,**C**,**E**,**F**): 130×.

**Figure 2:**
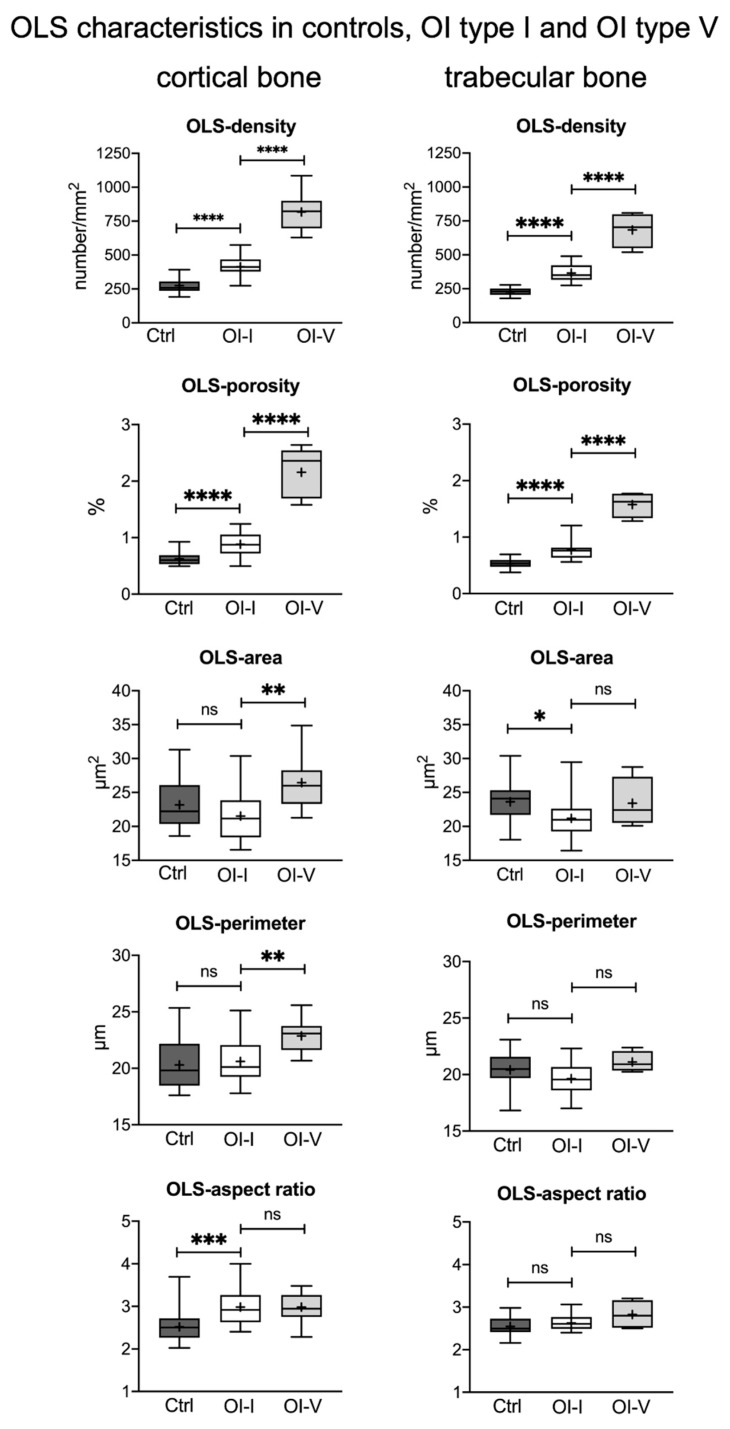
Results of OLS analysis: comparison of OLS characteristics in transiliac bone biopsy samples from children with OI type I (*n* = 19) versus controls (*n* = 24) and versus OI type V (*n* = 15, age: 8.7 ± 4 years-old) previously presented by Blouin et al. [49]. * *p* < 0.05, ** *p* < 0.01, *** *p* < 0.001, **** *p* < 0.0001; ns = not significant. The left column shows results in trabecular and the right column results in cortical bone. Data are presented using the box-and-whisker plot with the horizontal line in the middle showing median values and the top and bottom lines of the boxes showing the interquartile range. The + inside the boxes represents mean values and the horizontal lines above and below the boxes show maxima and minima.

Table 1 summarizes the results of the OLS analysis in trabecular and cortical bone from OI type I patients compared to controls. Figure 1 shows typical backscattered electron overview images of biopsy samples from cortical and trabecular bone from a healthy child (A, B, C) and a child with OI type I (D, E, F). OLS density and OLS porosity in OI type I were increased by +50.66% and +49.15%, respectively, in cortical bone and by +61.73% and +40.74%, respectively, in trabecular bone versus controls (all *p* < 0.001). Osteocyte lacunar size (OLS area and OLS perimeter) were found similar in cortical bone from controls and in OI. Only in trabeculae, the OLS area from OI type I was slightly smaller than in controls (relative decrease: 10.28%; *p* = 0.015). The OLS aspect ratio, reflecting the shape of the osteocytes, was similar in trabecular bone from OI type I and controls, while in cortical bone the value was increased; thus, the lacunae were more elongated in OI type I than in controls (relative increase: 16.8%; *p* < 0.001) (Figure 2, Table 1). Further comparisons of OLS in trabecular and cortical bone from the same patient showed that the OLS density as well as OLS porosity were lower in trabecular bone than in cortical bone in controls (relative decrease: 17.7%, *p* < 0.0001; relative decrease: 8.45%, *p* = 0.0014, respectively) and in OI (relative decrease: 11.7%, *p* < 0.0334; relative decrease: 13.64%, *p* = 0.0268, respectively). OLS area and OLS perimeter were similar in trabecular and cortical bone in controls and OI bone. The OLS aspect ratio was higher in cortical than in trabecular bone in OI type I samples, whereas there was no bone compartment related alteration in controls.

### 2.2. Osteocyte Lacunae Number, Size and Shape Are Correlated between Trabecular and Cortical Bone 

**Figure 3:**
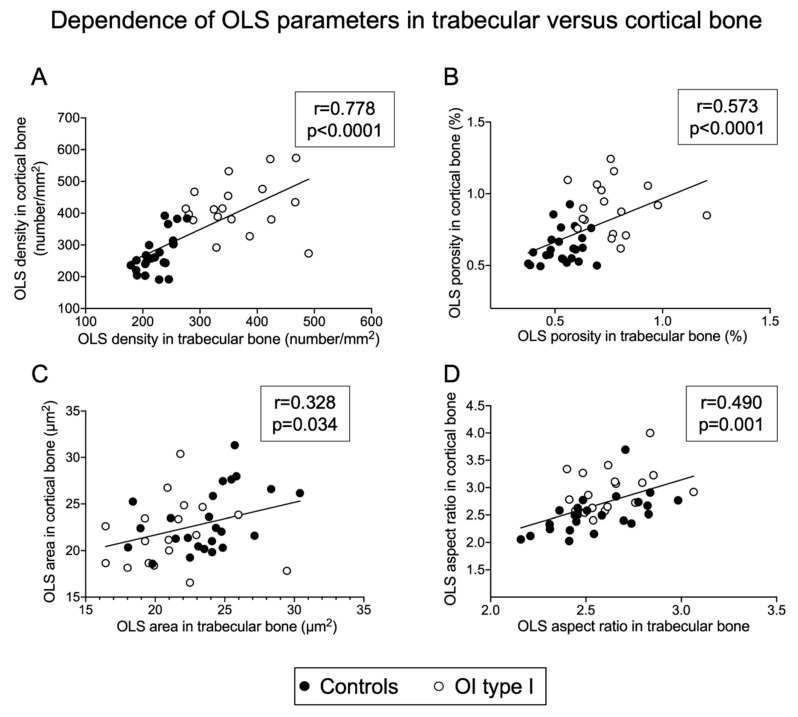
Dependency of OLS density (**A**), OLS porosity (**B**), OLS area (**C**) and OLS aspect ratio (**D**) in trabecular versus cortical bone (total sample analysis). Note that OLS density and OLS porosity in OI type I bone (white circles) is always higher than in controls (black circles). In contrast, there is no clear separation between both groups for OLS area and OLS aspect ratio.

There were high positive correlations for the OLS density (r = 0.778), and moderate positive correlations for the OLS porosity (r = 0.573, both *p* < 0.0001), as well as positive, but low correlations for the OLS area (r = 0.328, *p* = 0.0338) and the OLS aspect ratio (r = 0.490, *p* = 0.0001) between trabecular and cortical bone (Figure 3).

### 2.3. Osteocyte Lacunar Differences between Qualitative and Quantitative Mutations

No statistical differences in OLS characteristics were found between qualitative and quantitative mutations, except cortical OLS porosity that was significantly higher in qualitative mutations than in quantitative mutations (1.03% ± 0.16 versus 0.81% ± 0.17; *p* = 0.019) (see supplemental Table S1). 

### 2.4. Osteocyte Lacunae Density in OI Type I Is Markedly Lower Than in OI Type V

We further compared the present data with previously reported OLS characteristics in OI type V bone, which consists predominantly of primary bone with disordered collagen fibrils [19,46,49]. In comparison to OI type V, in OI type I, OLS density and OLS porosity were about 50% lower in both bone compartments. Moreover, the OLS area and OLS perimeter were significantly larger in cortical bone of OI type V than in OI type I (relative increase 22.8%, *p* = 0.0012; relative increase 10.92%, *p* = 0.0040, respectively), whereas there was no statistical difference in size of the trabecular bone of both forms of OI (Figure 2).

### 2.5. Association of Age with Osteocyte Lacunae Characteristics

**Figure 4:**
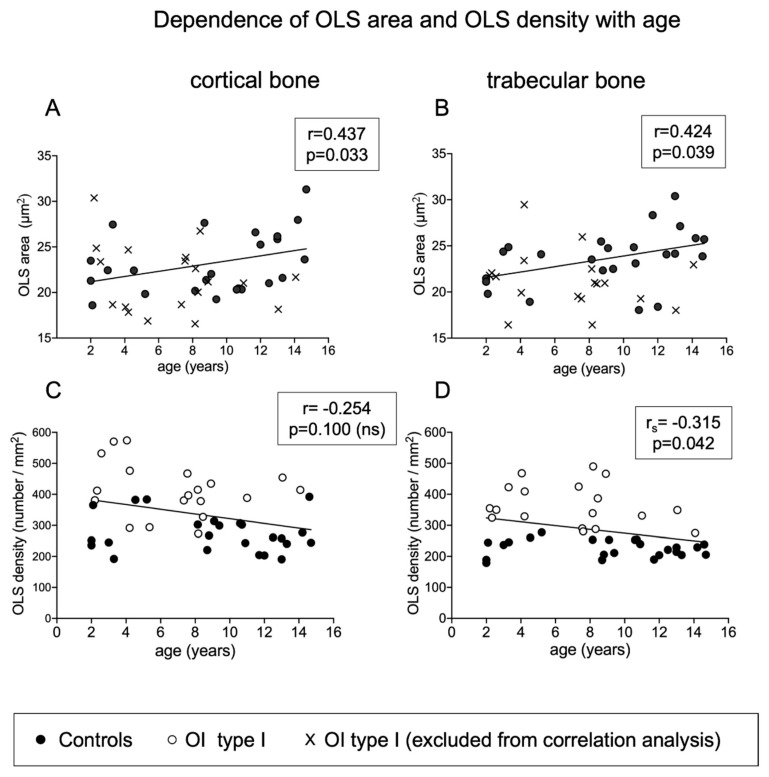
Dependency of OLS area (**A**,**B**) and OLS density (**C**,**D**) with age. OLS area increases slightly with age in controls but not in OI type I bone (*p* = 0.308 and 0.463, respectively, for cortical and trabecular bone). In contrast, OLS density is negatively correlated with age (total sample analysis). Note that at all ages, OLS density is higher in OI type I (white circles) than in controls (black circles).

In the control group, the OLS area increased significantly with age in cortical bone (r = 0.437, *p* = 0.033) as well as in trabecular bone (r = 0.424, *p* = 0.039). There was no significant relationship of age with OLS in OI bone. When all samples were pooled (thus, n = 43), OLS density decreased significantly with age (r_s_ = −0.375; *p* = 0.042) in trabecular bone but not in cortical bone. Generally, at all ages, OLS density was higher in OI than in controls (Figure 4).

### 2.6. Relationship between Osteocyte Lacunae Characteristics and Bone Histomorphometry Outcomes 

Differences in histomorphometric parameters between children with OI type I and controls were reported earlier [19,43,44]. Briefly, structural parameters such as cortical width and bone volume/tissue volume are significantly lower in OI, whereas bone surface-based formation indices such as osteoblast surface/bone surface, osteoid surface/bone surface, mineralizing surface/bone surface and bone formation rate/bone surface are significantly higher in OI. With regard to osteoblast function, OI patients have significantly lower mineral apposition rate and bone formation rate/osteoblast surface than healthy children. Osteoid thickness is also reduced in OI (All histomorphometric data are compiled in Supplemental Table S2).

Here we evaluated correlations of key bone histomorphometry parameters with OLS parameters in the OI type I (*n* = 19) and age-matched control group (*n* = 24) separately, as well as in the total cohort (n = 43). 

#### 2.6.1. Correlations of OLS Density and Porosity with Histomorphometric Parameters

In OI type I group, we observed moderate negative correlation between OLS porosity in trabecular bone and BV/TV (r = −0.532; *p* = 0.023). In the control group, we found low positive correlation between cortical bone OLS density with OS/BS (r = 0.407; *p* = 0.049) and low negative correlation with O.Th (r = −0.463; *p* = 0.023).

**Figure 5:**
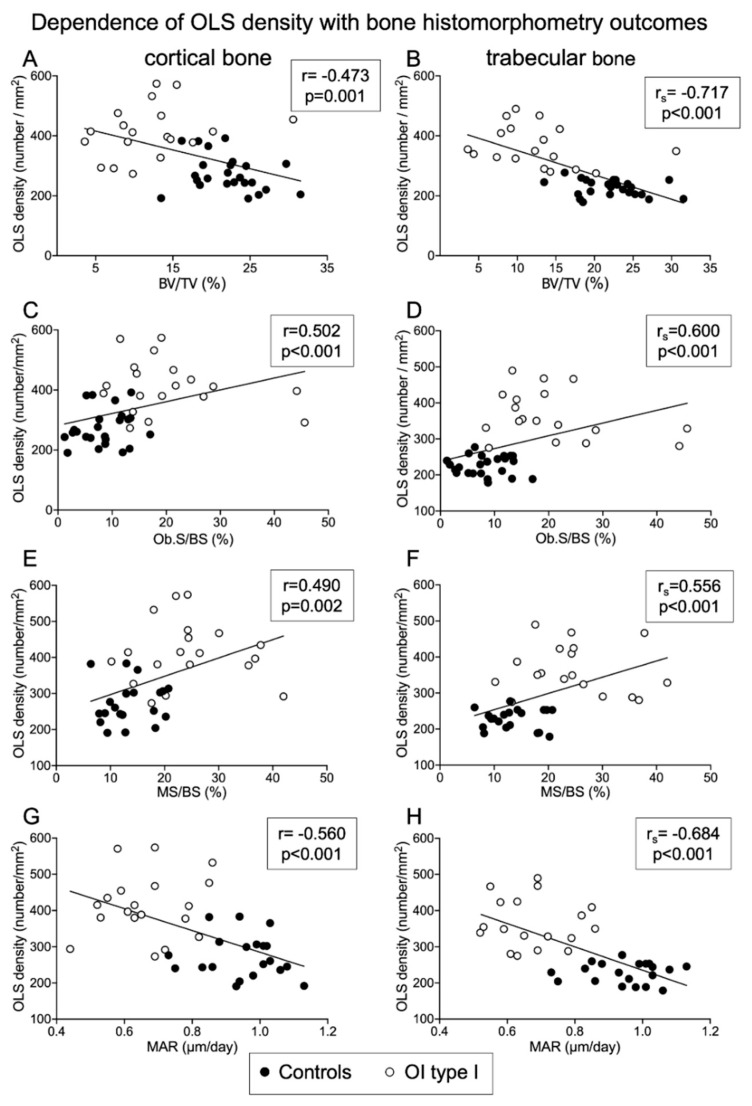
Dependency of OLS density with bone histomorphometric parameters (total sample analysis): OLS density is negatively correlated with structural parameters such as BV/TV (**A**,**B**), positively correlated with static (Ob.S/BS) and dynamic (MS/BS) surface-based bone formation indices (**C**–**F**), and negatively correlated with indices of osteoblast function as mentioned above (MAR) (**G**,**H**).

After pooling all samples, correlation analyses showed that OLS number; thus, OLS density and OLS porosity were negatively correlated with indices of bone volume and indices of osteoblast function but positively with surface-based bone formation indices. Some key correlations are summarized below for cortical and trabecular bone (see also Figure 5).

##### Negative Correlation of OLS Density and OLS Porosity with Structural Histomorphometric Parameters

Cortical width (Ct.Wi):

Trabecular bone: OLS porosity (r = −0.495; *p* = 0.001); OLS density (r_s_ = −0.375; *p* = 0.015).

Bone volume/tissue volume (BV/TV):

Trabecular bone: OLS density: (r_s_ = −0.717; *p* < 0001), OLS porosity (r = −0.635; *p* < 0.001); 

Cortical bone: OLS density (r= −0.473; *p* = 0.001); OLS porosity (r = −0.426; *p* = 0.004).

##### Negative Correlation of OLS Density and OLS Porosity with Indices of Osteoblast Function

Mineral apposition rate (MAR):

Trabecular bone: OLS density (r_s_ = −0.684; *p* < 0.001); OLS porosity (r = −0.537; *p* = 0.001); 

Cortical bone: OLS density (r = −0.560; *p* < 0.001); OLS porosity (r = −0.502; *p* = 0.001)

Bone formation rate/osteoblast surface (BFR/Ob.S): 

Trabecular bone: OLS density (r_s_ = −0.598; *p* = 0.001); OLS porosity (r_s_ = −0.582; *p* = 0.001); 

Cortical bone: OLS density (r_s_ = −0.465; *p* = 0.004); OLS porosity (r_s_ = −0.522; *p* = 0.001). 

Osteoid thickness (O.Th): 

Trabecular bone: OLS density (r_s_ = −0.521; *p* < 0.001); OLS porosity (r = −0.306; *p* = 0.049); 

Cortical bone: OLS density (r = −0.556; *p* < 0.001); OLS porosity (r = −0.459; *p* = 0.002).

##### Positive Correlation of OLS Density and OLS Porosity with Surface-Based Bone Formation Indices

Osteoblast surface/bone surface (Ob.S/BS): 

Trabecular bone: OLS density (r_s_ = 0.600; *p* < 0.001): OLS porosity (r = 0.476; *p* = 0.001); 

Cortical bone: OLS density (r = 0.502; *p* = 0.001); OLS porosity (r = 0.477; *p* = 0.001). 

Osteoid surface/bone surface (OS/BS): 

Trabecular bone: OLS density (r_s_ = 0.428; *p* = 0.005); OLS porosity (r = 0.409; *p* = 0.007); 

Cortical bone: OLS density (r = 0.384; *p* = 0.011).

Mineralizing surface/bone surface (MS/BS): 

Trabecular bone: OLS density (r_s_ = 0.556; *p* < 0.001); OLS porosity (r = 0.471; *p* = 0.003); 

Cortical bone: OLS density (r = 0.490; *p* = 0.002); OLS porosity (r = 0.355; *p* = 0.027).

All correlations obtained by pooling the data of the two cohorts were low to moderate, except the correlation of OLS density with BV/TV in trabecular bone, which was highly negative. There were no significant correlations of BFR/BS with OLS density or porosity.

#### 2.6.2. Correlation of OLS Area and OLS Perimeter with Histomorphometric Parameters 

Only correlations for trabecular bone were observed.

In OI type I:

OLS area and OLS perimeter with OS/BS (r = 0.587; *p* = 0.010 and r = 0.658; *p* = 0.003, respectively). 

OLS perimeter with Ob.S/BS: (r = 0.471; *p* = 0.049). 

In controls: 

OLS area and OLS perimeter with O.Th (r = 0.466; *p* = 0.022; r = 0.439; *p* = 0.032; respectively) 

**Figure 6:**
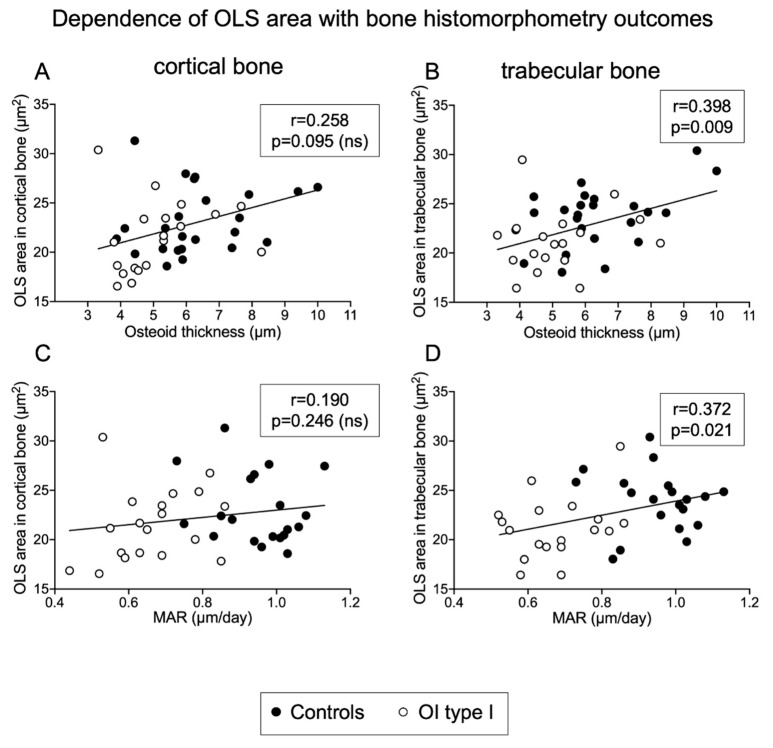
Dependency of OLS area with bone histomorphometry outcomes (total sample analysis): OLS area is positively correlated with osteoid thickness (**A**,**B**) and MAR (**C**,**D**), two parameters reflecting osteoblast function.

In the pooled data set: (Figure 6)

OLS area and OLS perimeter with O.Th (r = 0.398; *p* = 0.009 and r = 0.372; *p* = 0.015, respectively).

OLS area with BFR/Ob.S (r_s_ = 0.347; *p* = 0.041) and with MAR (r = 0.372; *p* = 0.021).

There were no significant correlations with Ct.Wi, BV/TV, Ob.S/BS, MAR or BFR/BS perimeters.

### 2.7. Bone Mineralization Density Distribution (BMDD) in OI Type I and Controls

The samples investigated in this work have been previously used to obtain reference BMDD curves for healthy children as well as children with OI type I [44,45]. These BMDD data were collected with an SEM equipped with a thermionic tungsten cathode, while the present data were collected using an SEM with a field emission cathode (FESEM). We have recently shown that to ensure a valid comparison between sample and reference, both have to be measured on the same SEM [50]. Therefore, we measured the BMDD of the transiliac biopsy samples from both OI patients and control cohort on the FESEM. The results are summarized in Table 2. The control group with n = 24 corresponds to the cohort defined in the present study as having the same age as the OI group, whereas in the control group with n = 50, adolescents until age of 20.9 years are included. There were no statistical differences for any BMDD parameter neither in trabecular nor in cortical bone between the two control groups. Furthermore, the BMDD parameters showed nearly no significant correlation with age (with exception of a moderate decrease of CaLow in trabecular and cortical bone (both, *p* = 0.03) and a slight increase of CaMean in cortical bone (*p* = 0.05). 

Compared to the control group (*n* = 24), OI type I had elevated CaMean (relative increase 3.79%; *p* = 0.0002) and CaPeak (relative increase 4.49%; *p* < 0.0001) and a nearly 3-fold higher CaHigh (relative increase 182%; *p* = 0.0005), similar CaLow (*p* = 0.6598) and lower CaWidth (relative decrease 13.65%; *p* < 0.0001) in trabecular bone. Next, in cortical bone, we observed an elevated CaMean (relative increase 7.23%; *p* < 0.0001) and CaPeak (relative increase 7.37%; *p* < 0.0001) and a highly elevated CaHigh (relative increase 130%; *p* = 0.0001), but a lower CaWidth (relative decrease 18.76%; *p* < 0.0001) and CaLow (relative decrease 31.1%, *p* = 0.0023). Differences of BMDD parameters between OI type I and the total control cohort revealed an even higher statistical significance due to the larger cohort sample size (*n* = 50) (Table 2). 

## 3. Discussion

In the present study, we measured bone biopsy samples from a previously presented study cohort of healthy children and children with OI type I in order to assess OLS characteristics [44,45]. The method was described earlier to characterize OLS in OI type V, a distinct form of osteogenesis imperfecta caused by a gain-of-function mutation in the *IFITM5* gene [49]. The typical histologic and microscopic features of OI type V are irregular lamellar orientation, exuberant primary bone formation and very elevated osteocyte density [46,47,48,49]. In contrast, OI type I represents the “classical” form of OI caused by collagen-gene mutations. At the tissue level, OI type I is characterized by regular bone lamellar orientation, although with thinner lamellae, increased osteoblast number, osteoid surface and decreased osteoblast function compared to healthy references [18,19].

In accordance with our previous studies of this OI population, where we have compared clinical data, bone histomorphometric parameters, bone mineralization density distribution, mineral particle size and the properties of the organic matrix, we found almost no significant differences in OLS parameters between quantitative and qualitative mutations [28,44,51]. The only exception was the OLS porosity in cortical bone that was modestly increased in subjects with qualitative mutations. This lack of mutation-specific alterations is a further indication that the specific collagen defect does not primarily cause the abnormalities of bone in OI with collagen-gene mutations. Hence, we pooled all OI type I samples (quantitative and qualitative mutations) and evaluated OLS parameters in the total study population.

We found several key differences in OLS characteristics between OI type I and controls:

- First, the OLS density is increased in OI both in cortical and trabecular bone. This means that the total amount of bone matrix per osteocyte is reduced in OI.
- Second, the increased OLS density correlates with increased surface-based bone formation parameters previously obtained by histomorphometry in the same samples [19,43,44]. This supports previous findings that the reduced matrix production rate of osteoblasts in OI is partially compensated by an increased number of active osteoblasts [19].
- Third, the OLS aspect ratio in cortical bone is increased in OI. Given that osteocytes typically align with the collagen direction, this surprisingly suggests the presence of more primary lamellar tissue in the cortex of OI type I bone.
- The final observation is that the OLS area (i.e., the mean lacunar size) positively correlates with osteoid thickness and mineral apposition rate, measured by histomorphometry. This correlation is not obvious to interpret, but we may speculate that the enhanced mineral deposition activity when osteoid is to be mineralized leads to an increase in OLS area, perhaps to provide additional mineral through osteocytic osteolysis.

The few published studies on bone tissue properties in OI consistently report increased number of osteocytes in OI patients and murine model of osteogenesis imperfecta [18,19,21,22,25,52,53,54,55,56]. Using SEM, Imbert L. et al. analyzed cortical bone samples from corrective surgery from five children with OI and three controls [22]. They found a 50% increase in osteocyte density in OI, which is in accordance with our data. However, the reported values are much higher in controls (395.8 ± 15.8/mm^2^) and in OI (640.8 ± 195/mm^2^) than in our samples, which might be related to the specific bone site (lower extremity). Indeed, Zimmermann et al. reported a similar high osteocyte number in pediatric femoral bone [54]. In contrast, the OLS density in trabecular bone in our reference cohort is very close to the values reported by Jandl et al. in trabeculae of iliac bone from six children [57]. However, we found a smaller OLS area in trabecular bone (23.63 ± 3.01 µm^2^ versus 30.71 ± 4.25 µm^2^) and in cortical bone (23.19 ± 2.24 µm^2^ versus 31.70 ± 7.76 µm^2^). This could be due to the difference in number of analyzed samples, a wider age range (2–14.7 years in our control group versus 2–8 years) and/or age-related variation of osteocyte lacunae in our control group (but not in the OI cohort). Additionally, technical settings (grey-levels settings and size thresholds to discriminate osteocyte lacunae from the surrounding matrix) might be different to the aforementioned work.

A further interesting observation is that osteocyte lacunae density is higher in cortical than in trabecular bone, which is in accordance with a higher cortical remodeling activity during skeletal growth [57,58,59]. Osteocyte density in cortical bone, however, appears rather variable. In the report of Jandl et al., for example, osteocyte density was found to be twice as high than in the present study (537 ± 127 mm^2^ versus 274.65 ± 60.14 mm^2^) [57]. The reason for this discrepancy is possibly that we excluded areas of very porous primary woven bone from our measurements. Primary woven bone and primary lamellar bone are formed during skeletal growth, as part of a process described as modeling drift, in which new bone apposition and bone resorption occur on opposite surfaces of the two cortical plates to allow the ilium to increase in width [60,61]. Primary bone is generally formed as lamellar sheets that run parallel to the periosteal surface before being remodeled into osteonal bone [60,61,62]. However, in situation of growth spurt, loosely organized woven bone is laid down. This bone tissue is characterized by randomly arranged collagen I fibrils and elevated osteocyte density [45,60,63]. We have previously shown that woven bone can be distinguished from lamellar bone on qBEI images by the typical increased cellularity and elevated mineralization compared to the adjacent osteonal remodeled bone [45,49]. Hence, areas of woven bone with OLS density about 500.6 ± 149/mm^2^ (data not shown) were observed in half of the biopsy samples in the control group. This rather high incidence is consistent with the observation that primary bone formation is the highest in children and adolescents until 14 years of age, which corresponds exactly to the age range of our control group [61]. However, half of the analyzed biopsy samples from the control group did not contain any primary woven bone, and therefore, to make the data more comparable, we excluded it from the present evaluations. 

Of note, no primary woven bone was observed in biopsy samples from our OI study cohort. It is interesting that the OLS aspect ratio, a measure of the elongation of osteocyte lacunae, was the highest in the cortices of OI bone. Osteocyte lacunae tend to align their longest axis parallel to the preferred collagen orientation [64,65]. This would indicate that in cortical bone from children with OI the relative amount of parallel lamellar bone formed by periosteal apposition is higher than in cortical bone of controls. In other terms, the remodeling process of primary into secondary osteonal bone is delayed or disturbed in OI, which is in agreement with previous observations [18,19]. Conversely, a smaller OLS aspect ratio reflects secondary remodeled bone in osteons and trabeculae, in which there is no predominant collagen orientation within the 2D image plane.

The correlation between elevated osteoblast number on the bone surface and increased OLS density within the bone matrix seems logical at a first glance. It has to be noted, however, that the calculated correlation coefficient values were low to moderate (varying between 0.3 and 0.7), suggesting that the elevated number of osteocytes in OI bone cannot be solely explained by the increased osteoblast pool in OI bone. This relation is possibly complicated by the fact that osteocytogenesis and the osteocyte transcriptome are also broadly dysregulated in OI [10,19]. A crucial step at the osteoblast-to-osteocyte transition is the downregulation of collagen and the upregulation of matrix degrading enzymes and markers for dendrite formation and intercellular communication [3,66,67]. In osteocytes from OI mouse models, however, collagen expression was found to be upregulated, which was interpreted as an attempt to counteract the insufficient bone formation [10]. Hence, it seems very likely that not only osteoblast number, but also osteoblast and osteocyte dysfunction, play an important role in regulating the number of osteocytes that are finally incorporated within the bone matrix. 

Moreover, osteoid thickness is increased in the control group mirroring together with the increased mineral apposition rate, the vigorous osteoblast activity during skeletal growth [43,68]. The positive correlation between the OLS area with osteoid thickness and with mineral apposition rate in trabecular bone may indicate that some osteocytic osteolysis, and thus, some demineralization of the perilacunar matrix, occurs in deeper bone regions, possibly to provide additional calcium to the mineralizing surfaces. In fact, osteocytic osteolysis is observed in metabolic situations of calcium and vitamin D deficiency, which probably also occur transiently during growth [69]. 

It is interesting to note that the OI-associated bone fragility appears very different than aging-associated osteoporosis, which is associated with decreased lacunar density, and thus, with a less effective mechanosensitive response [8,70,71,72,73]. However, and despite the limited age range in our study population, we also observed a significant decrease of OLS density with age. Nevertheless, at any age, OLS density was always higher in OI bone than in controls. A further notable difference is that in age-related osteoporosis the bone matrix becomes under-mineralized, whereas in OI bone the matrix is over-mineralized [74]. In line with our previous results, the current analyses, performed on a field emission scanning electron microscope, showed significantly higher bone matrix mineralization and lower heterogeneity in mineralization in OI versus controls. A more mineralized and therefore stiffer bone matrix may further reduce the mechano-sensitivity of the osteocyte network. 

It should be underlined that our study detects osteocyte lacunae within the mineralized matrix instead of living osteocytes. Thus, osteocyte viability within the lacunae cannot be evaluated by this method. Conversely, we characterized only unmineralized OLS and not hypermineralized lacunae reflecting dead osteocyte remnants in the bone matrix that have subsequently been mineralized. Previously, Boyde A et al. identified few “occluded” lacunae in pediatric controls as well as OI bone samples but less than in adult bone, in line with more recent studies showing osteocyte death is accelerated during aging [4,18,71]. Elevated number of hypermineralized osteocytes in OI bone was not reported [18]. 

In addition to the comparison of OLS characteristics between OI bone and healthy references, we also presented reference BMDD values for both sample cohorts obtained on an SEM equipped with a field emission cathode. In line with previous findings, bone matrix mineralization in OI was found significantly higher than in healthy controls [12,44,75]. Again, this confirms the hypermineralization of bone matrix that is common to all types of OI. Furthermore, the obtained BMDD reference parameters for OI as well as for the healthy controls showed no age dependence (with the only exception of a mildly significant dependence of CaLow in both compartments and CaMean in the cortical compartment). This allows to average all BMDD parameters to obtain age-independent reference BMDD for both cohorts. 

The major strength of our study is that we provide new data on osteocyte lacunae characteristics in 19 children with OI and 24 controls and correlated them with histomorphometry data from the same sample block Next, we present new pediatric reference BMDD values, obtained with a FESEM microscope, from a cohort 50 healthy individuals, encompassing the age range from 2 to 20.9 years. 

We acknowledge that the study has certain limitations. As noted previously, the bone samples from the control cohort were obtained during surgical procedures for localized conditions, and therefore, although none of the study subject showed signs of bone disorders, they were not strictly healthy. Neither pubertal stages nor serum markers of bone metabolism were evaluated when the biopsy were obtained [43,61]. Thus, although the sex distribution was similar in both groups (males 62.5% in controls and 68.4% in OI type I, *p* = 0.684), we did not evaluate the differences between males and females to avoid possible blurring because of different pubertal statuses. However, previous studies reported that size and/or density of osteocyte lacunae do not differ between men and women [9,72,73].

## 4. Patients and Methods

### 4.1. Study Cohort

Bone biopsy sample were obtained from previous histomorphometric studies by Glorieux, FH et al. [19,43]. The study protocol was approved by the Ethics Committee of the Shriners Hospital for Children in Montreal, Canada and informed consent was obtained in each instance from the subject and/or a legal guardian as appropriate. In the present study we re-used the residual sample blocks to perform quantitative back-scattered electron microscopy with a high-resolution field emission scanning electron microscope to assess 2D OLS analysis and to determine bone mineralization density distribution (BMDD) [44,45].

The OI study cohort consisted of 19 patients (male, *n* = 13; female, *n* = 6) with OI type I. Bone biopsy samples were obtained before the start of any osteotropic medication. The total age range of the patients at the time of obtaining the biopsy was from 2.2 to 14.1 (median: 7.6) years. Six of them were diagnosed having qualitative mutations and 13 quantitative mutations. All clinical, genetic and histomorphometric data were presented previously [44].

For comparison with OI data, we used a control cohort consisting of 24 healthy children (male, n = 15; female, n = 9, total age range 2.0 to 14.7 years; median: 10.0 years) that did not differ in age from the OI study population (*p*-value: 0.067, Mann–Whitney U test). This cohort was presented previously and is part of a larger population [43,44,45]. The bone biopsies were obtained during surgery for various orthopedic conditions including lower limb deformities, scoliosis, clubfeet and other problems that require corrective surgery (exostoses, cubitus valgus, equinovarus of the foot). All of them were ambulatory, had normal renal function as assessed by measurement of serum creatinine and no evidence of any metabolic bone disorder [43,44,60].

Moreover, to establish new reference BMDD values, we extended our bone mineralization density distribution analyses (BMDD) by quantitative backscattered electron imaging (qBEI) to a larger cohort of 50 bone biopsy samples obtained from healthy individuals, encompassing the age range from 2 to 20.9 years (median: 12.95 years). These biopsy samples were obtained and used previously to establish normative data for iliac bone histomorphometry and BMDD with a scanning electron microscope (SEM) equipped with a tungsten hairpin cathode (thermionic electron emission, Zeiss DSM 962, Zeiss Oberkochen, Germany) ([43,45]). 

### 4.2. Sample Preparation for qBEI Measurements

The sectioned bone surfaces from the residual polymethylmetacrylate-embedded samples blocks were ground with sandpaper with increasing grid-number and then polished with diamond grains (size down to 1 µm). Finally, the sample surface was carbon-coated by vacuum-evaporation (Agar SEM carbon-coater; Agar Scientific Stansted, UK) [76].

### 4.3. Quantitative Backscattered Electron Imaging (qBEI)

qBEI was performed with a field emission scanning electron microscope (FESEM, Zeiss SEM SUPRA 40) equipped with a Schottky field emission electron gun with zirconium envelope. The measurements were performed at 20kV with a working distance of 10 mm and a scan speed of 90 s per frame [50]. The intensity of backscattered electrons from the surface of a bone biopsy sample is proportional to the local mean atomic number. A calibration of the FESEM with carbon and aluminum prior to the measurement allows to quantitatively assess the local calcium content in the sample. Brightness and contrast of the detector are tuned in order to obtain a gray level of 25 ± 1 for carbon and 225 ± 1 for aluminum. Thus, the obtained 8-bit grey-level images can be translated into mineralization maps given in calcium weight percent (weight % Ca) [50,76,77]. Osteocyte lacunae sections are then evaluated on these images (see Section 4.4). Moreover, the grey-level histogram (denoted bone mineralization density distribution-BMDD) providing the frequency distribution of each calcium content is analyzed (bin width of 0.17 weight %Ca) (see Section 4.5). 

### 4.4. Osteocyte Lacunae Sections Analysis

qBEI images were captured from the cortical and cancellous part of the bone at a nominal magnification of ×130 (pixel size 0.9µm/pixel) and had a grey-level resolution of 256 grey-level steps. 

In order to perform OLS analysis, we used the grey-level pictures of both cortices and the trabeculae that we obtained with the FESEM [76,78]. These pictures were then used for a 2D characterization of osteocyte lacunae sections by using a custom-made macro in ImageJ software (version 1.52; NI, Bethesda, MD, USA). Setting a threshold based on a fixed grey-level (5.2 weight %Ca), we discriminated lacunae from surrounding mineralized bone matrix [49]. Furthermore, we set a size threshold between 5 µm^2^ and 200 µm^2^ to distinguish between OLS and osteonal channels. In accordance with our previous qBEI analyses, we observed highly porous primary bone on the periosteal side of the cortex in half of the biopsy samples of the control group [45]. These areas were excluded from our evaluations as number and size of the lacunae in primary bone differ significantly from secondary bone [49].

OLS were characterized by five parameters similarly as described previously for OI type V bone [49]:

- OLS density (OLS number/mm^2^): the number of OLS/(mineralized bone matrix area + OLS total area).
- OLS porosity: (%) OLS total area/(mineralized bone matrix area + OLS total area).
- OLS area (µm^2^): mean value of the OLS areas per sample (total OLS area divided by OLS number).
- OLS perimeter (µm): mean value of the OLS perimeters per sample.
- OLS aspect ratio: mean value of the OLS aspect ratio per sample. The OLS aspect ratio is a measure for the shape of the OLS. It is given by the ratio of the long to the short half-axis of a fitted ellipse to the section. A value of 1 indicates a perfect circle, while increasing values indicate an increasing elongated shape. OLS with aspect ratio values >10 were excluded from the analysis.

Number of lacunae analyzed per sample (values are given as median and interquartile range [25%; 75%]):

In trabecular bone: OI type I: 264 [111; 651]; controls: 574 [478; 752].

In cortical bone: OI type I: 1403 [878; 1808]; controls: 1097 [776; 1404].

### 4.5. Bone Mineralization Density Distribution (BMDD)

For evaluation of the BMDD, the entire cross-sectional area of the transiliac bone sample was scanned with a 65x nominal magnification, corresponding to a resolution of 1.76 µm per pixel. From the obtained 8-bit image, a frequency histogram of grey levels was obtained. The grey scale was then transformed into a mineralization scale given in weight % calcium (weight %Ca) resulting in BMDD, which measures the amount of bone that is mineralized with a certain calcium content.

The BMDD is characterized by five parameters [76,77]:

- CaMean: the average calcium concentration (weighted mean);
- CaPeak: the most frequently occurring calcium concentration (the position of the peak of the BMDD);
- CaWidth: the width of the BMDD distribution (full-width at half-maximum), reflecting the heterogeneity in matrix mineralization;
- CaLow: the percentage of bone material mineralized below 18.20 weight %Ca, which corresponds to the 5th percentile of the reference BMDD in adult trabecular bone;
- CaHigh: the percentage of bone matrix having a mineral content above 26.86 weight %Ca, corresponding to the 95th percentile of the reference BMDD in adult trabecular bone.

The results are given separately for cancellous and cortical bone. The results for cortical bone are calculated as the arithmetic mean of both cortices for each sample [45,50].

### 4.6. Bone Histomorphometry

Bone histomorphometry outcomes were previously published [19,43]. 

For correlation analysis, the following parameters were evaluated in the present study: cortical width (Ct.Wi), bone volume/tissue volume (BV/TV), osteoid thickness (O.Th), osteoid surface/bone surface (OS/BS), osteoblast surface/bone surface (Ob.S/BS), mineralizing surface/bone surface (MS/BS), mineral apposition rate (MAR), bone formation rate/bone surface (BFR/BS) and bone formation rate/osteoblast surface (BFR/Ob.S).

### 4.7. Statistical Analyses

We compared OLS, BMDD parameters and bone histomorphometry parameters in the OI cohort (n = 19) and in the control group (n = 24) that did not differ in age. We further compared the OLS parameters of OI type I patients obtained in the present study with OLS parameters of data obtained previously in OI type V patients by unpaired T-test [49].

Characteristics of samples of OI and healthy children were described using frequencies and percentages for categorical variables and means and standard deviation (SD) for continuous variables. In case of variables which did not conform to a normal distribution, medians and interquartile ranges (IQR) were computed. We assessed the distribution of each parameter via normality plots and by Shapiro–Wilk test on a total sample and also on subsamples of OI and healthy children. 

For group comparisons (OI type I vs. healthy children, OI type I qualitative vs. quantitative mutation), we used an independent samples T-Test or Mann–Whitney U test for continuous variables and a Chi-square test for categorical variables, as appropriate. For comparison of OLS in trabecular and cortical bone from the same patient we used a paired T-test. 

Comparison of BMDD parameters between the OI study cohort and the control groups (*n* = 24 and *n* = 50, encompassing the total young reference age from 2.0 to 20.9 years) were calculated by unpaired T-test for CaMean and CaPeak and by Mann–Whitney U test for CaWidth, CaLow and CaHigh. Two-sided *p*-values of 0.05 or less were considered statistically significant for all findings. Throughout the manuscript, we report exact *p* values where appropriate.

For the exploration of an association between two continuous variables, we calculated either Pearson’s correlation coefficient (r) or Spearman’s rank correlation coefficient (r_s_), according to the normality of variable distribution. Variables which were not normally distributed were log-transformed or square root-transformed and tested again for normality. If the transformed data were normally distributed, we proceeded with parametric correlation, otherwise with non-parametric. As low correlation we considered coefficient values between 0.30–0.50; as moderate 0.50–0.70; and as high 0.70–0.90. 

Analyses were done in IBM SPSS version 26 (IBM Corp. Released 2019. IBM SPSS Statistics for Windows, Version 26.0. Armonk, NY, USA: IBM Corp) and in GraphPad Prism version 9.1.0 for Windows (GraphPad Software, San Diego, CA, USA, www.graphpad.com accessed on 10 April 2021).

## 5. Conclusions

The aim of this study was to show that the correlation of standard bone histomorphometry data with the analysis of OLS characteristics (osteocyte lacunae number, area and shape) in a bone biopsy provides new insights in the relation of bone forming osteoblasts and their descendants, the osteocytes. In particular, it was shown that OLS density in OI is highly increased and this increase correlates significantly with surface-based bone formation parameters obtained by histomorphometry. In contrast, OLS area is slightly decreased in trabecular bone from children with OI. Due to the high number of samples investigated, the obtained results are statistically highly robust. 

We also present reference BMDD data for bone from children with OI type I and from healthy adolescents. In contrast to previously published references that were obtained using an SEM with a thermionic tungsten cathode, in the current work, we used a field emission scanning electron microscope. The found results are consistent with the earlier published BMDD data showing no age dependency in the investigated age range as well as a hypermineralization of OI bone compared to healthy references.

## Figures and Tables

**Table 1:** Results of OLS analysis in trabecular and cortical bone from OI type I patients compared to controls.

	Trabecular Bone			Cortical Bone			Trabecular Versus Cortical Bone	
OLS parameters:	OI type I (*n* = 19) *	Controls (*n* = 24)	*p*-value	OI type I (*n* = 19) *	Controls (*n* = 24)	*p*-value	OI type I (*p*-value)	Controls (*p*-value)
Density (number/mm^2^)	365.60 (67.73)	226.00 (26.75)	<0.0001	413.80 (86.25)	274.65 (60.14)	<0.0001	0.0334	<0.0001
porosity (%)	0.76 [0.64; 0.81]	0.54 [0.48; 0.60]	<0.0001	0.88 [0.72; 1.06]	0.60 [0.53; 0.69]	<0.0001	0.0268	0.0014
area (µm^2^)	21.20 (3.16)	23.63 (3.01)	0.0150	21.53 (3.66)	23.19 (3.34)	0.1278	0.5944	0.5211
perimeter (µm)	19.65 (1.51)	20.42 (1.50)	0.1070	20.61 (2.21)	20.30 (2.09)	0.6368	0.1110	0.7512
aspect-ratio	2.63 (0.18)	2.55 (0.22)	0.2185	2.92 [2.63; 3.27]	2.50 [2.27; 2.72]	0.0003	0.0020	0.2522

OLS parameters were evaluated in 19 OI-I biopsy samples and 24 age-matched reference biopsy samples. * In one OI-I sample, no trabecular bone was present. All other samples contained two cortices and trabeculae. Values are presented as mean (±standard deviation) or median [interquartile range: 25%; 75%].

**Table 2:** BMDD in controls and OI type I.

Trabecular Bone				
BMDD parameters	Controls (*n* = 24)	Controls (*n* = 50)	OI type I (*n* = 19) *	Diff. OI type I versus controls (*n* = 50)
CaMean (weight % calcium)	22.45 (0.73) 22.45 [22.25; 22.80]	22.48 (0.73) 22.60 [22.12; 22.96]	23.30 (0.59) 23.28 [22.92; 23.64]	*p* < 0.0001
CaPeak (weight % calcium)	23.40 (0.75) 23.40 [23.09; 23.92]	23.39 (0.70) 23.57 [23.01; 23.79]	24.45 (0.61) 24.44 [24.05; 24.83]	*p* < 0.0001
CaWidth (**Δ** weight % calcium)	3.91 (0.47) 3.81 [3.64; 3.99]	3.76 (0.50) 3.64 [3.47; 3.99]	3.39 (0.20) 3.29 [3.29; 3.51]	*p* = 0.003
CaLow (% bone area)	6.46 (1.90) 5.95 [5.39; 6.95]	6.14 (2.21) 5.57 [4.78; 6.80]	6. 55 (1.65) 6.19 [5.31; 7.41]	*p* = 0.1475
CaHigh (% bone area)	2.04 (1.93) 1.53 [0.69; 2.48]	1.82 (1.64) 1.52 [0.62; 2.22]	5.77 (4.79) 4.29 [2.00; 8.48]	*p* < 0.0001
**Cortical Bone (calculated as arithmetic mean of both cortical plates)**				
BMDD parameters	Controls (*n* = 24)	Controls (*n* = 50) **	OI type I (*n* = 19) *	Diff. OI type I versus controls (*n* = 50)
CaMean (weight % calcium)	21.84 (1.18) 22.02 [21.41; 22.84]	21.86 (1.15) 22.17 [21.05; 22.76]	23.42 (0.71) 23.50 [22.97; 24.07]	*p* < 0.0001
CaPeak (weight % calcium)	22.66 (1.29) 22.88 [22.38; 23.57]	22.67 (1.21) 22.96 [22.10; 23.48]	24.33 (0.70) 24.52 [23.92; 24.87]	*p* < 0.0001
CaWidth (**Δ** weight % calcium)	4.32 (0.76) 4.16 [3.75; 4.98]	4.23 (0.67) 4.07 [3.73; 4.68]	3.36 (0.29) 3.38 [3.21; 3.64]	*p* < 0.0001
CaLow (% bone area)	9.63 (6.76) 7.28 [5.07; 10.78]	9.19 (6.18) 6.86 [5.06; 11.48]	4.98 (0.87) 5.02 [4.38; 5.68]	*p* < 0.0007
CaHigh (% bone area)	1.47 (1.15) 1.01 [0.66; 2.26]	1.37 (1.21) 1.01 [0.44; 1.89]	4.75 (3.61) 3.52 [2.05; 6.49]	*p* < 0.0001

Data are presented as mean (SD) or median with interquartile range [25%; 75%]. * In one OI-I sample, no trabecular bone was present. All other samples contain two cortices and trabeculae. ** In two control samples, only one cortical plate was available and were, therefore, discarded.

## Data Availability

Data is contained within the article and supplementary material.

## Supplementary Materials

The following are available online at https://www.mdpi.com/article/10.3390/ijms22094508/s1, Table S1: Comparison of OLS between quantitative and qualitative mutations: Clinical, genetic and histomorphometric bone mineralization and properties of the organic matrix were reported elsewhere [28,51,77]. Statistically significant differences among the two subgroups are shown in bold, Table S2: Bone histomorphometric outcomes of the present study population. The present study groups with the corresponding bone histomorphometry data are part of a larger cohorts presented previously [19,43,44]. Bone histomorphometry outcomes for the biopsy samples used in the present study are compiled below. Data are presented as mean (SD) or median with interquartile range [25%; 75%].
