# Supplementary material for: Increased Osteocyte Lacunae Density in the Hypermineralized Bone Matrix of Children with Osteogenesis Imperfecta Type I
Source: Int J Mol Sci. 2021 Apr 26;22(9):4508. doi: 10.3390/ijms22094508 (PMC8123504; doi:10.3390/ijms22094508)
Supplement: Supplementary file 1 [file ijms-22-04508-s001.zip › ijms-1153697-supplementary.pdf]

International Journal of
Molecular Sciences

MDPI

Table S1: Comparison of OLS parameters between OI Type I quantitative and qualitative mutations:

OLS Parameters	OI qualitative mutation (n=6)	OI quantitative mutation (n=13)	p-value	test
**Cortical bone**				
**density (number/mm²)**	433.44 (58.27)	404.72 (97.27)	0.516	t-test
**porosity (%)**	**1.03 (0.16)**	**0.81 (0.17)**	**0.019**	**t-test**
**area (μm²)**	23.41 [21.13-25.48]	18.67 [17.99-23.65]	0.087	MWU
**perimeter (μm)**	20.47 [19.99-22.60]	19.63 [18.11-22.32]	0.244	MWU
**aspect-ratio**	2.86 (0.28)	3.04 (0.45)	0.401	t-test
**Trabecular bone**				
**density (number/mm²)**	345.59 (66.74)	375.55 (68.84)	0.393	t-test
**porosity (%)**	0.74 [0.62-0.83]	0.77 [0.65-0.82]	0.682	MWU
**area (μm²)**	21.49 (2.47)	21.05 (3.55)	0.789	t-test
**perimeter (μm)**	19.59 (1.38)	19.67 (1.63)	0.922	t-test
**aspect-ratio**	2.50 [2.47-2.60]	2.63 [2.55-2.82]	0.102	MWU

Table S2: Comparison of Bone histomorphometric parameters between Controls and OI Type I:

Bone histomorphometry variables	Controls (n=24)	OI type I (n=19)	P value	Difference %	test
		**Structural parameters**			
**CORE Width**	7.25 (2.03)	3.94 (1.61)	**<0.001**	-45.66	t-test
**Ct.Wi**	769.00 [643.2-1081.5]	539.00 [427.0; 765.0]	**0.002**	-29.91%	MWU
**BV/TV**	22.14 (4.24)	12.17 (6.28)	**<0.001**	-45.03%	t-test
**Tb.Th**	130.91 (24.38)	109.79 (29.42)	**0.014**	-16.13%	t-test
**Tb.N**	1.70 (0.21)	1.07 (0.35)	**<0.001**	-37.06%	t-test
**BS/BV**	16.75 (3.14)	18.99 (4.91)	0.08	+13.37%	t-test
**BS/TV**	3.61 (0.45)	2.12 (0.74)	**<0.001**	-41.27%	t-test
		**Static parameters of bone formation**			
**O.Th**	6.32 (1.57)	5.13 (1.33)	**0.006**	-18.83%	MWU
**OS/BS**	27.81 [21.30; 34.26]	39.87 [27.31; 61.69]	**0.004**	+43.37%	MWU
**OV/BV**	2.89 (1.20)	4.40 (2.56)	**0.025**	+52.25%	t-test
**Ob.S/BS**	8.15 [5.19; 11.91]	17.76 [13.84; 24.58]	**<0.001**	+117.91%	MWU
**Ob.S/OS**	29.19 (14.30)	46.31 (11.84)	**<0.001**	+58.65%	t-test
		**Dynamic parameters of bone formation**			
**MS/BS**	13.50 (4.52)	24.39 (8.74)	**<0.001**	+80.67%	t-test
**MS/OS**	48.10 (17.90)	58.57 (18.05)	0.077	+21.77%	t-test
**MAR**	0.95 (0.11)	0.66 (0.12)	**<0.001**	-30.53%	t-test
**Aj.AR**	0.45 (0.15)	0.39 (0.12)	0.162	-13.33%	t-test
**Mlt**	15.14 (4.64)	14.46 (5.36)	0.678	-4.49%	t-test
**Omt**	6.78 (1.72)	7.82 (1.88)	0.078	+15.34%	t-test
**BFR/BS**	47.19 (17.74)	59.16 (23.78)	0.082	+25.37%	t-test
**BFR/BV**	76.04 (36.24)	108.58 (44.66)	**0.017**	+42.79%	t-test
**BFR/ObS**	531.28 [409.53; 905.89]	310.53 [240.97; 354.62]	**<0.001**	-41.55%	MWU
		Bone resorption parameters			
**ES/BS**	15.93 [12.78; 17.98]	18.18 [15.27; 20.25]	0.163	+14.12%	MWU
**Oc.S/BS**	0.88 [0.58; 1.52]	1.19 [0.98-1.52]	0.123	+35.23%	MWU
**N.Oc/B.Pm**	0.31 [0.15; 0.41]	0.39 [0.30-0.43]	**0.047**	+25.81%	MWU
